# Exudative Retinal Detachment Without Choroidal Effusion After Glaucoma Surgery in Sturge-Weber Syndrome

**DOI:** 10.7759/cureus.97089

**Published:** 2025-11-17

**Authors:** Alejandro M Perez, Natalie DeBolske, Basil K Williams Jr., Peter Chang

**Affiliations:** 1 Ophthalmology, Bascom Palmer Eye Institute, Miami, USA; 2 Vitreoretinal Surgery and Ocular Oncology, Bascom Palmer Eye Institute, Miami, USA; 3 Glaucoma, Bascom Palmer Eye Institute, Miami, USA

**Keywords:** baerveldt, glaucoma, metoprolol, propranolol, retinal detachment, sturge-weber syndrome, trabeculotomy

## Abstract

An eight-day-old full-term infant with early-onset glaucoma associated with Sturge-Weber syndrome (SWS) underwent a trabeculotomy combined with a stage 1 Baerveldt glaucoma implant. After five years of stability, the patient developed increased intraocular pressure and axial length in the affected eye, necessitating the activation of the Baerveldt glaucoma implant with concurrent scleral windows. Postoperatively, the patient developed an inferior exudative retinal detachment, which was initially monitored, followed by administration of intravitreal metoprolol. This resulted in the complete resolution of the retinal detachment within six months. We report the first case of postoperative exudative retinal detachment in the absence of choroidal effusion in a child with SWS-related glaucoma. The clinical course raises the possibility that beta-blockers could potentially mitigate certain complications following glaucoma surgeries in these patients. Larger, prospective studies are necessary to generate high-level evidence.

## Introduction

Sturge-Weber syndrome (SWS) is a rare, congenital neurocutaneous disorder characterized by a combination of neurological, dermatological, and ocular manifestations [1]. It is typically associated with a facial capillary malformation, commonly known as a port-wine birthmark (PWB), and leptomeningeal vascular malformations in the brain, detectable via contrast-enhanced magnetic resonance imaging (MRI) [2]. The syndrome is caused by post-zygotic somatic mosaic mutations in the *GNAQ* gene, leading to abnormal vascular development [1,2]. SWS has an estimated prevalence of one in 20,000 to one in 50,000 live births, with no preference for race or gender [1]. Approximately half of all patients have ocular involvement. Of these ocular manifestations, glaucoma is the most common and challenging, affecting 30-70% of cases, followed by choroidal hemangioma, which is seen in 40-50% of cases [3]. Glaucoma associated with SWS and PWB is present in a bimodal distribution, with some patients showing early onset (0-3 years) and others developing the condition later in life, up to 41 years of age [4].

The pathophysiology of glaucoma in SWS remains incompletely understood, though it is primarily attributed to abnormal development of the iridocorneal angle [3]. Characteristic features of the angle in SWS include flat iris insertion, prominent vascular loops at the iris root, and blood within Schlemm's canal [3,5-7]. Given the vascular nature of SWS, it is unsurprising that the literature suggests a less favorable response to angle surgery, indicating that the underlying pathology may extend beyond the trabecular meshwork to involve more distal structures, such as increased episcleral venous pressure [3]. Postoperative complications, including serous choroidal effusions and suprachoroidal hemorrhage, have been reported following glaucoma filtering surgeries, particularly in eyes with abnormal vascular dynamics such as those seen in SWS [8-10]. However, the development of an exudative retinal detachment in the absence of choroidal effusion is rare and highlights the unusual nature of this presentation.

Herein, we present a case of SWS-associated glaucoma complicated by postoperative exudative retinal detachment without choroidal effusion, managed with systemic and intravitreal beta-blockers.

## Case presentation

**Figure 1:**
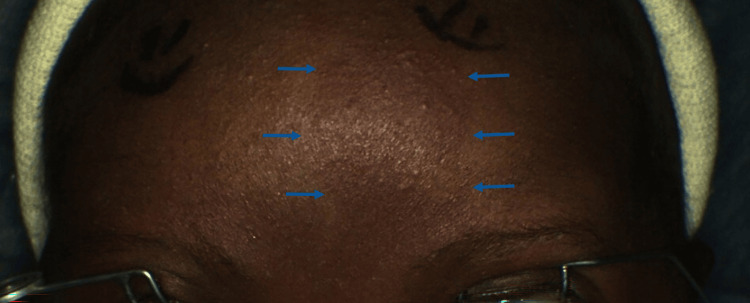
Clinical photograph showing a midline facial port-wine birthmark outlined with blue arrows.

**Figure 2:**
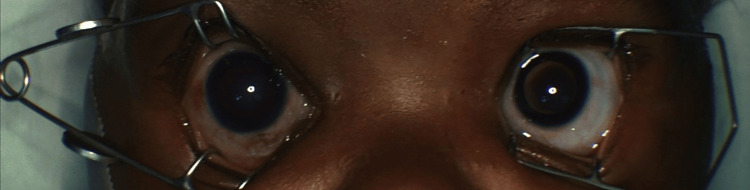
Examination under anesthesia showing an increased corneal diameter in the right eye compared to the left, along with generalized corneal haze in the right eye.

An eight-day-old full-term infant was referred to the clinic with concerns about a cloudy right cornea and the presence of a PWB. The parents reported an uncomplicated pregnancy and delivery; however, a midline facial birthmark and a hazy right cornea were observed by the attending neonatologist on the first day of life, as seen in Figures 1, 2, respectively. The infant was suspected to have SWS; the initial neuroimaging performed elsewhere was reportedly negative. The patient was discharged from the hospital on the seventh day of life on dorzolamide-timolol fixed combination. The parents also mentioned a history of reactive airway disease but denied any history of stridor or underlying airway malformations. They also did not observe any abnormal behaviors or seizures in the infant.

During the initial clinic exam, the infant grimaced at bright light and was unable to cooperate with other types of visual acuity and intraocular pressure measurements. An exam under anesthesia (EUA) was promptly performed. During EUA, the intraocular pressure (IOP) was measured using a Tono-Pen® handheld tonometer (Reichert, Inc., Depew, New York, United States) under anesthesia, with readings of 18 mmHg in the right eye and 10 mmHg in the left eye, while on dorzolamide-timolol (2%/0.5%), one drop in the right eye twice daily.

**Figure 3:**
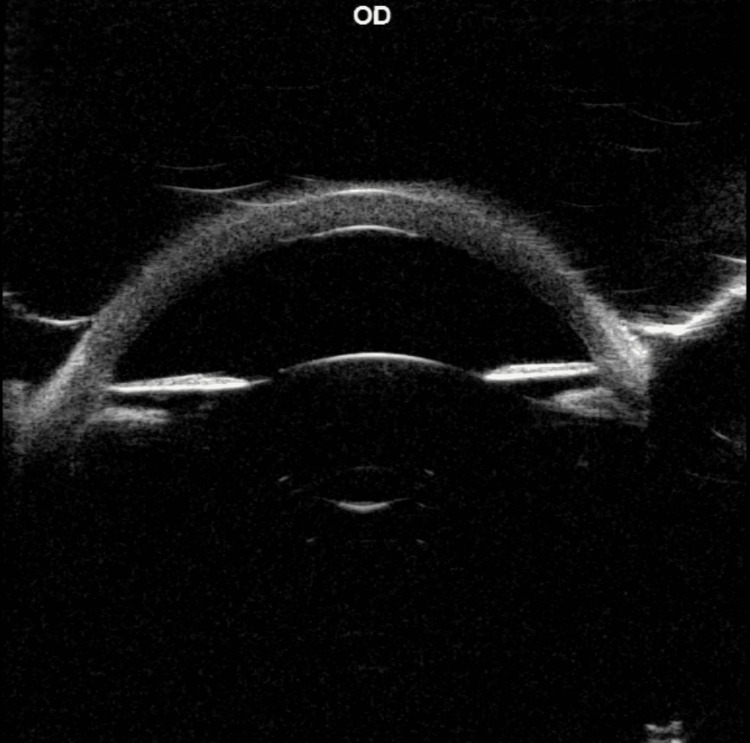
Ultrasound biomicroscopy image displaying a well-formed anterior chamber, open angles, and a normal lens structure in the right eye.

**Figure 4:**
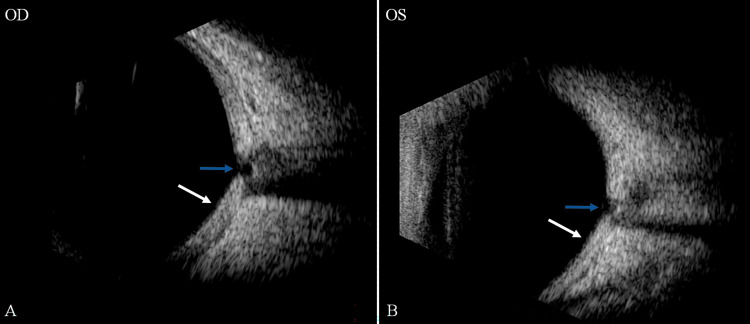
B-scan echography of the right (A) and left (B) eyes showing mild vitreous opacities and diffuse thickening of the ocular coats in the right eye (white arrows) compared with the left. Bilateral moderate optic disc cupping is present, more pronounced in the right eye (blue arrows).

On exam, the right eye had an increased corneal diameter of 11.5 mm (horizontal and vertical), whereas the left eye measured 10.0 mm (horizontal and vertical). There was generalized right corneal haze (Figure 2). Ultrasound biomicroscopy showed a well-formed anterior chamber, open angles, and a normal lens (Figure 3). B-scan echography showed mild vitreous opacities and diffuse thickening of the ocular coat in the right eye, with bilateral moderate optic disc cupping, more pronounced in the right eye (Figure 4). Upon discharge from the clinic, topical latanoprost was prescribed for the right eye. Oral propranolol therapy was initiated in anticipation of possible surgery [11].

**Figure 5:**
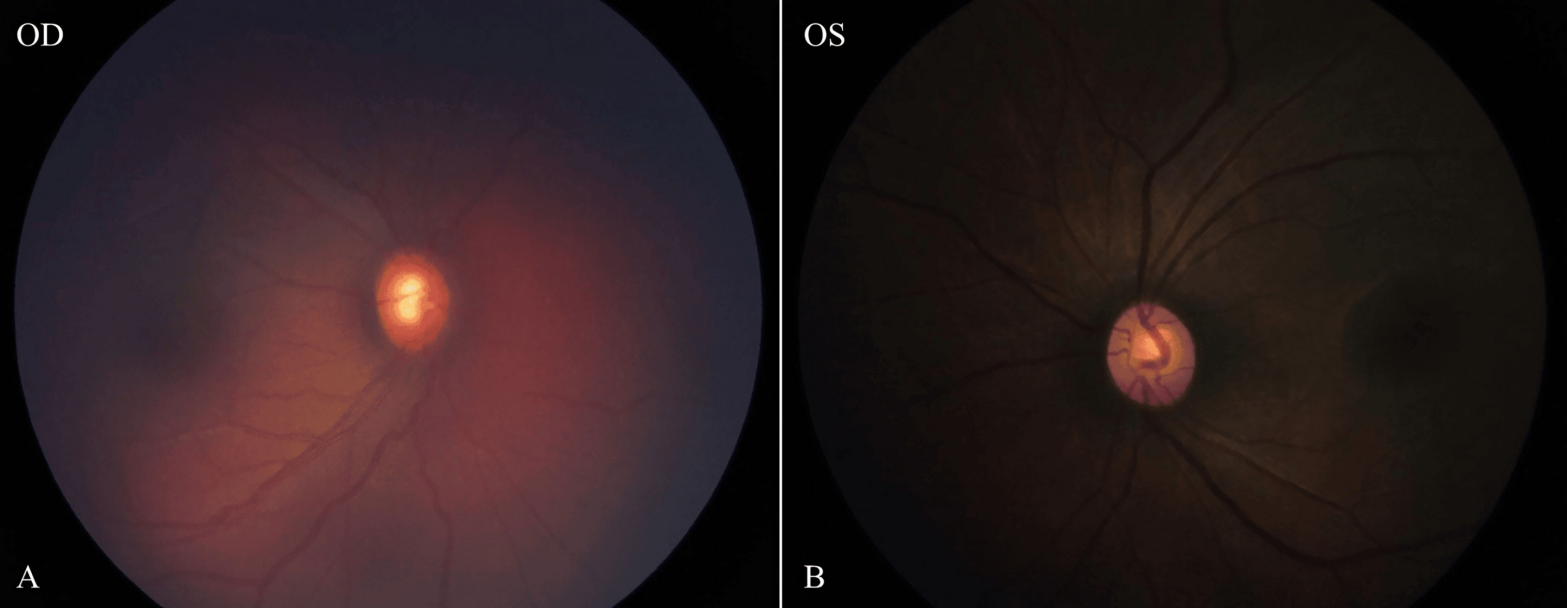
Fundus photographs of the right (A) and left eye (B) showing diffuse corneal edema and optic nerve cupping in the right eye, with a normal appearance in the left eye.

One week later, the child underwent an exam under anesthesia, which confirmed elevated IOP (28 mmHg on the right and 12 mmHg on the left), diffuse corneal edema, and optic disc cupping more in the right than the left eye (Figure 5). A circumferential trabeculotomy using an illuminated microcatheter was performed, and a stage 1 Baerveldt® 250 drainage device (Johnson & Johnson Vision, Irvine, California, United States) was placed in the inferonasal quadrant of the right eye. Postoperatively, the patient did well without any immediate complications.

Over the subsequent five years, the visual acuity in the right eye ranged between 20/400 and count fingers, with a mean intraocular pressure of 25 mmHg, skewing closer to 30 mmHg in the later periods. Eventually, five years after the initial procedure, the right eye IOP became uncontrolled medically, and the optic disc demonstrated increased concentric cupping. The previously placed Baerveldt glaucoma implant was activated by inserting the tube into the inferotemporal anterior chamber, accompanied by prophylactic sclerotomies. The patient received systemic propranolol (2 mg/kg/day) beginning one week before the procedure, and a planned six-week course after the procedure [11].

**Figure 6:**
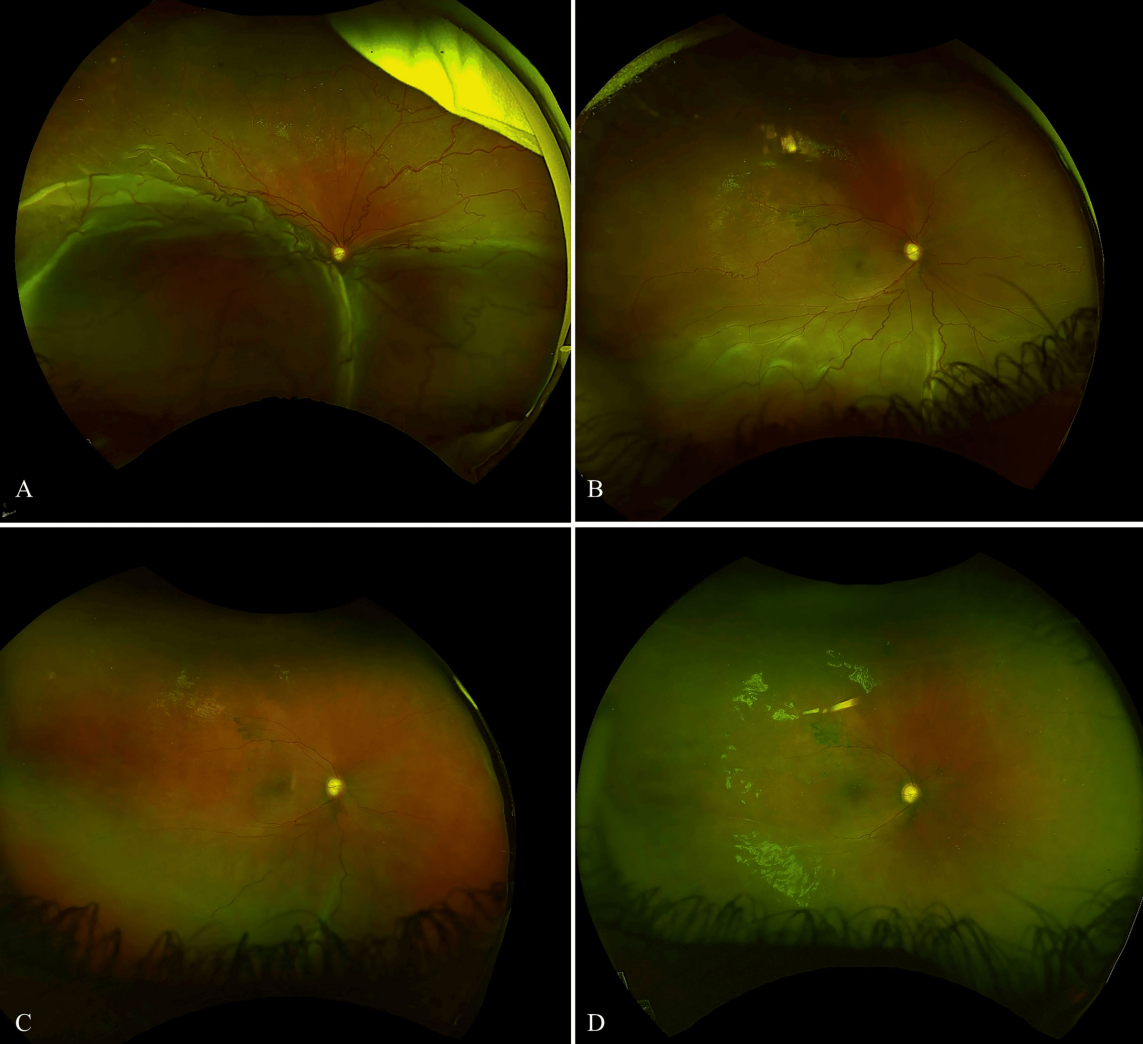
Fundus photographs over time showing the progression of retinal detachment recovery. (A) Postoperative day 1, with a significant inferior exudative retinal detachment. (B) Postoperative day 30, showing considerable improvement, though inferior subretinal fluid remains. (C) Postoperative month 4, demonstrating continued improvement with persistent subretinal fluid. (D) Postoperative month 6, where the retinal detachment has fully resolved.

**Figure 7:**
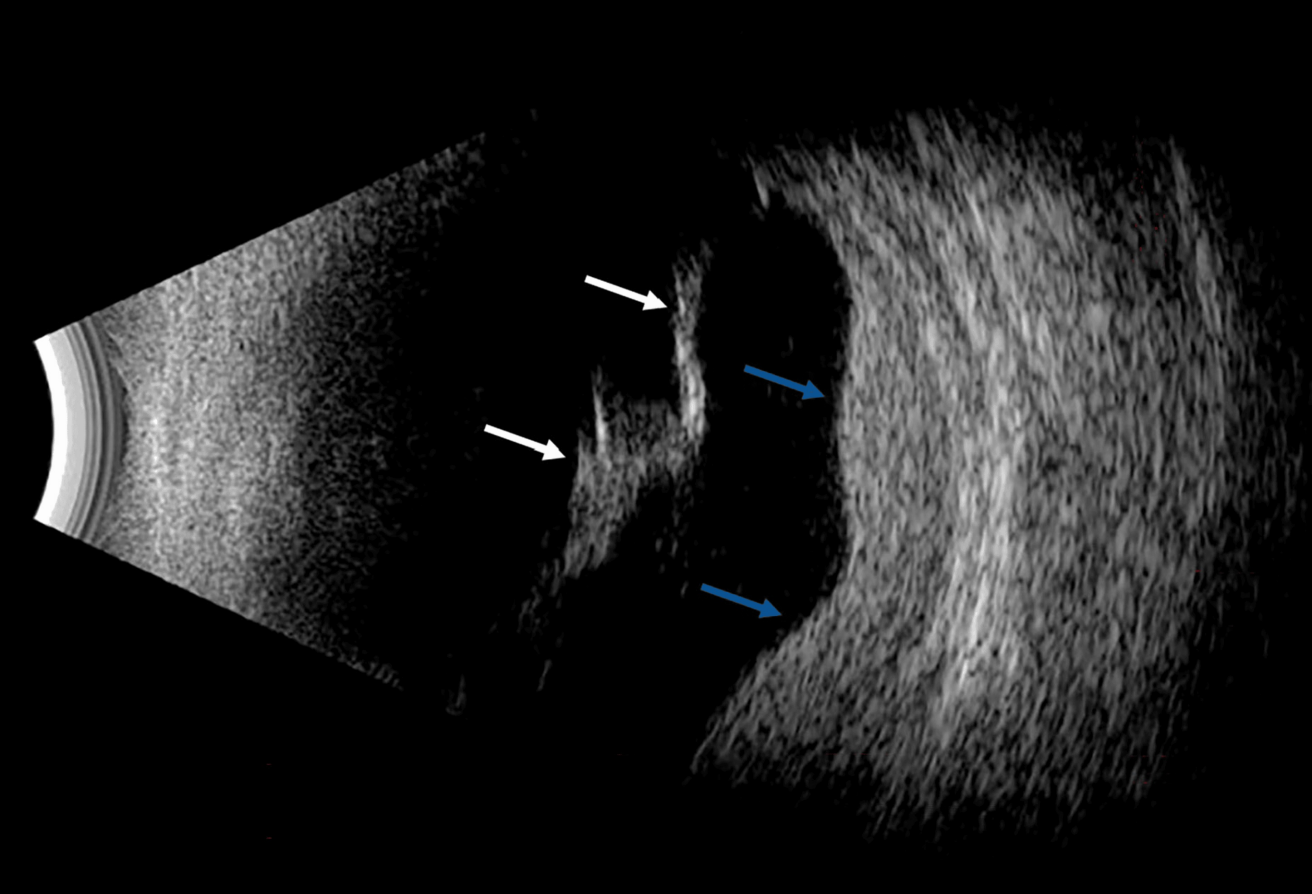
B-scan ultrasonography of the right eye demonstrating an exudative retinal detachment without evidence of choroidal effusion (white arrows). The guitar-shaped contour of the hyperreflective outer layer corresponds to a thickened choroid (blue arrows), which further increased in thickness postoperatively.

On the first postoperative day, the patient had an IOP of 18 mmHg, a formed and deep chamber, and a clear cornea. On the posterior segment exam, a significant, macula-involving, inferior exudative retinal detachment was visible (Figure 6A); no choroidal detachment was noted on B-scan ultrasound (Figure 7).

The patient was urgently referred to a pediatric retina specialist. The detachment was initially managed with close observation. By postoperative day 30, the detachment had considerably improved, though inferior subretinal fluid was still present (Figure 6B). Four months after surgery, there was persistent subretinal fluid. Treatment strategies and options were discussed with the family, and they preferred pharmacologic treatment to expedite the resolution of the retina detachment and decrease travel burden. Thus, an intravitreal metoprolol injection was administered (50 µg/0.05 mL) (Figure 6C). By the sixth postoperative month, the retinal detachment had entirely resolved (Figure 6D), without further intervention, and the visual acuity remained hand motion. The patient's IOP remained controlled on dorzolamide-timolol (2%/0.5%).

## Discussion

Early-onset glaucoma in patients with SWS presents significant management challenges, especially when the condition is refractory to medical therapy, as seen in our case. Historically, patients who present before the age of one year are typically treated with goniotomy or trabeculotomy, given that their glaucoma is often due to trabecular dysgenesis [12]. Our patient initially had well-controlled IOP following angle surgery (with concurrent stage 1 tube shunt plate placement), but ultimately required glaucoma drainage device activation; this two-stage approach decreases the risk of hypotony and allows concurrent scleral window creation at the time of IOP lowering.

Complications following angle surgery in patients with SWS are well-documented, often emerging as early as the first postoperative day. These complications frequently include flat anterior chambers accompanied by choroidal detachments [12]. Serous choroidal effusions, characterized by smooth lobes of elevated retina and choroid, are typically limited by the vortex veins [8]. According to the literature involving adult patients, the incidence of such effusions after trabeculectomy ranges from 7.9% to 18%, and with a glaucoma drainage device implant, it ranges from 11.7% to 15% [8,13-16]. While these complications are common, we took specific precautions in our approach to prevent them.

Serous retinal detachments (SRDs), on the other hand, are rare, with only a few cases documented in the literature. Diffuse choroidal hemangiomas (DCH), commonly associated with SWS, are considered a significant risk factor for developing SRDs, particularly in the context of an abnormal choroid. Choroidal thickening exerts mechanical pressure on the retina, disrupting its typical structure and function [17]. This disruption impairs fluid drainage from the retinal vessels, eventually leading to degeneration of the retinal pigment epithelium (RPE) [17,18]. Once the RPE is compromised, it can no longer effectively pump fluid from the subretinal space to the choroid, disturbing the fluid balance within the retina and contributing to the development of SRDs [17]. Another potential source of subretinal fluid in our case could have been the increased inflammation of the abnormal choroidal vasculature following surgery. Nonetheless, the prophylactic scleral windows were intended to prevent the accumulation of subretinal fluid and the formation of SRDs.

This risk is further heightened by fluctuations in IOP during surgical procedures, which can cause the DCH to expand, allowing fluid to seep into the subchoroidal and subretinal spaces. For instance, one reported case involved an exudative retinal detachment that occurred after an Ahmed valve implantation in a SWS patient with a DCH in the absence of hypotony [19]. Another case highlighted an SRD in a SWS patient shortly after starting latanoprostene bunod, which supports the role of inflammatory mediators in the pathogenesis of SRDs [17]. Mueller and Chang, in their report, also did a literature review and found that only six out of 35 cases of patients with PWB reported an ipsilateral SRD following a bleb-forming procedure [17]. Our case is unique because no SRDs were reported without a previously noted diffuse choroidal hemangioma at the time of this search.

The use of beta blockers for treating conjunctival hemangiomas is well-documented in the literature, showing effectiveness in improving outcomes for periocular infantile and conjunctival hemangiomas [20,21]. Beta-blockers work by non-selectively antagonizing β-adrenergic receptors, leading to vasoconstriction. This effect can often be seen immediately, with a change in the hemangioma's color and a gradual reduction in its size [21,22]. Studies have demonstrated that non-specific beta-blockers, like propranolol, can induce apoptosis in capillary endothelial cells, contributing to hemangioma's shrinkage [21-23]. Additionally, beta-blockers have been shown to modulate angiogenic peptides such as vascular endothelial growth factor, hypoxia-inducible factor 1-alpha, and fibroblast growth factor, which are crucial in hemangioma development [22,24]. By downregulating these peptides, beta-blockers help halt endothelial proliferation.

Similarly, case studies have reported the use of systemic and intravitreal beta-blockers, such as propranolol and metoprolol, to aid in the healing of exudative retinal detachment caused by diffuse choroidal hemangiomas or circumscribed choroidal hemangiomas [25-27]. In our case, oral propranolol was administered to reduce the risk of choroidal effusion, which is considered standard practice for patients undergoing surgery for SWS-related glaucoma [11]. Additionally, intravitreal metoprolol was efficacious in resolving the retinal detachment. The rationale behind this approach is that in cases of chronic serous retinal detachment, there is a risk of developing retinal folds and scarring, which can significantly impair long-term vision. While the exudative retinal detachment improved with observation alone, intravitreal metoprolol was administered to accelerate fluid resolution, minimizing the risk of long-term vision loss.

While current clinical trials are still evaluating the off-label use of intravitreal metoprolol tartrate for patients with circumscribed choroidal hemangiomas, early evidence suggests that it is safe and may offer a viable treatment option for subretinal fluid secondary to choroidal hemangiomas [25,28]. In our case report, the creation of prophylactic scleral windows and the postoperative injection of intravitreal metoprolol may have facilitated the resolution of the exudative retinal detachment. However, further studies are needed to generate high-quality evidence to support the use of beta blockers in managing similar complications.

## Conclusions

We have presented the first case of postoperative exudative retinal detachment without choroidal effusion in a child with SWS-related glaucoma. This case highlights the potential benefits of incorporating beta-blockers into the management strategy for complex glaucoma with associated retinal complications. The positive outcome observed suggests that intravitreal metoprolol may represent a potential adjunct in this setting. Further studies are needed to evaluate its safety and efficacy.
